# Ferroptosis in Intracerebral Hemorrhage: A Panoramic Perspective of the Metabolism, Mechanism and Theranostics

**DOI:** 10.14336/AD.2022.01302

**Published:** 2022-10-01

**Authors:** Chenxiao Lu, Changwu Tan, Hongfei Ouyang, Zhuohui Chen, Zhouyi Yan, Mengqi Zhang

**Affiliations:** ^1^Department of Neurology, Xiangya Hospital, Central South University, Changsha, China.; ^2^National Clinical Research Center for Geriatric Disorders, Xiangya Hospital, Central South University, Changsha, 410008, China.; ^3^Xiangya School of Medicine, Central South University, Changsha, 410031, China

**Keywords:** ferroptosis, intracerebral hemorrhage, iron metabolism, lipid peroxidation, antioxidant

## Abstract

Iron is one of the most crucial elements in the human body. In recent years, a kind of programmed, non-apoptotic cell death closely related to iron metabolism-called ferroptosis- has aroused much interest among many scientists. Ferroptosis also interacts with other pathways involved in cell death including iron abnormality, the cystine/glutamate antiporter and lipid peroxidation. Together these pathological pathways exert great impacts on intracerebral hemorrhage (ICH), a lethal cerebrovascular disease with a high incidence rate and mortality rate. Furthermore, the ferroptosis also affects different brain cells (neurons and neuroglial cells) and different organelles (mitochondria and endoplasmic reticulum). Clinical treatments for ferroptosis in ICH have been closely investigated recently. This perspective provides a comprehensive summary of ferroptosis mechanisms after ICH and its interaction with other cell death patterns. Understanding the role of ferroptosis in ICH will open new windows for the future treatments and preventions for ICH and other intracerebral diseases.

## 1. Introduction

Cell death is critical in diverse physiological and pathological processes. It is an indispensable mechanism of hemostasis to maintain tissue morphology and function [1, 2]. Cell death can be categorized as regulated cell death (RCD) and accidental cell death (ACD). ACD usually occurs after accidental physical and chemical stimulation while RCD has a precise regulation through biological signaling pathways [3]. Ferroptosis, a form of regulated non-apoptotic cell death characterized by iron metabolic disorders [4], has been widely studied in recent years [5]. Typically, ferroptosis is related to iron metabolism disorders, glutathione (GSH)-dependent antioxidant defenses inactivation and lipid peroxidation. When iron overloads in a cell, reactive oxygen species (ROS) will accumulate and attack DNA, proteins, and lipids. Moreover, the GSH-dependent antioxidant defenses against ROS collapse. The fierce attack of ROS and the failed defense together predispose the cell to ferroptosis [6].

Intracerebral hemorrhage (ICH), a devastating and typical type of stroke with high mortality and morbidity, affects more than 15 million people worldwide annually [7, 8]. When a fragile vessel ruptures and blood flows into the brain, hemoglobin, which contains iron, is released from lysed red blood cells. Then, the concentrations of iron in the blood and between tissues change, followed by an imbalance of iron metabolism, the formation of lethal ROS and lipid peroxidation [8]. Researchers have found various forms of cell death after ICH, including apoptosis, necrosis and autophagy in animal models. However, these types of cell death fail to fully explain the brain damage in the early stages of ICH [9]. The basic mechanisms of ferroptosis have been elaborated in many studies and ferroptosis has been found to participate in the brain damage after ICH, which replenishes the understanding of ICH pathophysiology. Studies found that reducing iron accumulated with iron chelators after ICH, which induced iron toxicity and contributed to early brain injury [9, 10]. Recently, a novel lipophilic iron-chelating agent called pyridoxal isonicotinoyl hydrazine (PIH) has been reported to alleviate excess iron-induced cytotoxicity, which might help to prevent neuroinflammatory injury and promote neural functional recovery after ICH [11]. Furthermore, ferroptosis was found to interact with other cell death patterns and together they mediate the pathological process after ICH [12]. For instance, programmed necrosis, typical of inflammatory cytokines release and a necroinflammatory response, can be caused by excess iron-induced cytotoxicity and thus connected with ferroptosis. Ferroptosis can also cause secondary damages in different brain cells (including glial cells and neurons) and in different organelles (including mitochondria and endoplasmic reticulum) [14,15]. Recently, a variety of drugs targeting ferroptosis have emerged, paving the way for the treatment of ICH.

In this paper, we summarize and elucidate the potential ferroptosis pathways in the brain injury after ICH and the specificity of ferroptosis in different brain cells and organelles. We further discuss the treatment targeting ferroptosis, hoping to provide novel ideas for future ICH therapy.

## 2. Iron metabolism in the human body

Iron, one of the most essential trace elements in the human body, is a necessary component for maintaining normal physiological functions, such as oxygen transport, cellular respiration, energy production and immune function maintenance, etc.

**Figure 1.**
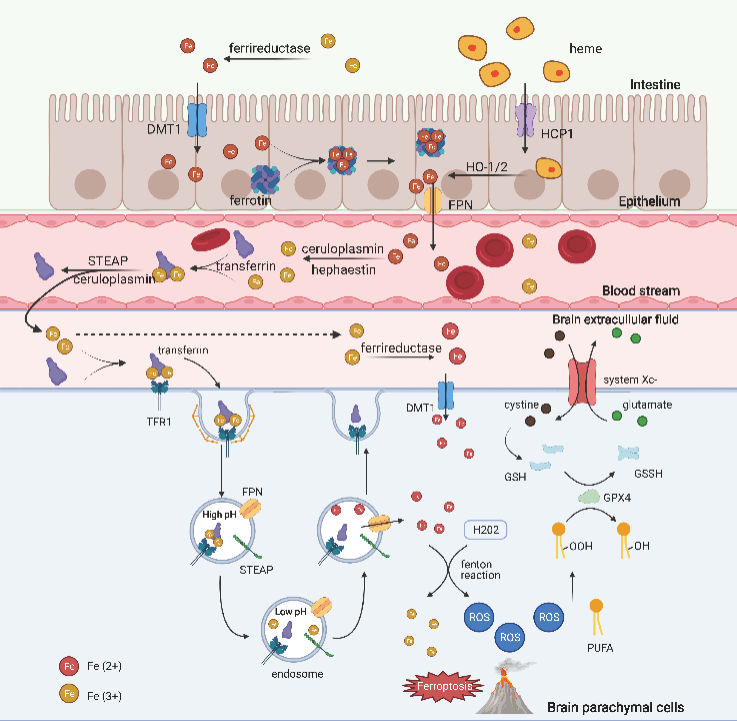
**The absorption of iron from the digestive tract into the circulatory system**. Fe^3+^ in the extracellular fluid is reduced to Fe^2+^ and transported into the cells (intestinal epithelial cells or other cells in the intestinal) via DMT1. HCP1 transports heme into the cells, releasing Fe^2+^. Most of the Fe^2+^ is then bound to ferritin, and the ferritin-Fe^2+^ complex slowly releases Fe^2+^, which is excreted out of the cell via FPN. The excreted Fe^2+^ is oxidized to Fe^3+^ by ceruloplasmin or hephaestin and bound to transferrin for storage and circulation to various body tissues. In the brain, binding of transferrin-Fe^3+^ complex and the membrane protein TFR1 of brain parenchymal cells induces endocytosis and then releases Fe^3+^. Fe^3+^ is reduced to Fe^2+^ and released into the cytoplasm via FPN. NTBI can enter the cell via DMT1. Fe^2+^ in the cytoplasm produces ROS via Fenton reaction, which allows PUFA to form lipid peroxides and affects cellular metabolism. Lipid peroxides react with GSH to form non-toxic alcoholic compounds under the action of GPX4. System Xc- transports cystine into the cytosol, promotes GSH production and reduces the amount of ROS. Abnormalities in any of the above processes of this pathway may induce ferroptosis. DMT1, divalent metal transporter 1; FPN, ferroportin; GPX4, glutathione peroxidase 4; GSH, glutathione; HCP1, heme carrier protein 1; H_2_O_2_, hydrogen peroxide; HO-1/2, heme oxygenase 1 or 2; NTBI, non-transferrin-bound iron; PUFA, polyunsaturated fatty acid; ROS, reactive oxygen species; STEAP, six-transmembrane epithelial antigen of prostate; TFR1, transferrin receptor 1.

Most of the iron in food is trivalent iron (Fe^3+^), which is not easily absorbed by the intestinal mucosa. When Fe^3+^ is reduced to divalent iron (Fe^2+^) by ferrireductase [13, 14], it will be transported into the cell through the divalent metal transporter 1 (DMT1) in the apical membrane of mucosal cells [15, 16]. Another transporter of Fe^2+^ into the cell, Heme carrier protein 1 (HCP1), is also located in the apical membrane and it transports dietary heme into the cytosol [17, 18]. The heme transferred into the cell releases Fe^2+^ with the action of heme oxygenase 1 or 2 (HO-1 or HO-2) [19, 20]. Both DMT1 and HCP1 are important participants in the uptake of iron in many tissues, including the brain. Most of the iron absorbed into the mucosa of the intestine bond with ferritin, an iron-sequestering protein that can carry up to 4,500 iron atoms [21], and together they form a Ferritin-iron complex which prevents the toxic effects of excess iron on cells [22]. Fe^2+^ is slowly released from the complex and is transported to the circulatory system via ferroportin (FPN), the most well-studied channel for iron excretion by far, on the side of the basement membrane [23]. Hepcidin, a peptide highly expressed in the liver [24], binds to FPN, oxidizes Fe^2+^ to Fe^3+^ [25], induces FPN endocytosis and degradation, and further blocks the efflux of iron [26] (Fig. 1).

Iron also participates in many metabolic pathways in the brain. However, due to the unique barrier structures in the brain-blood-brain barrier (BBB) and the blood-cerebrospinal fluid barrier, the entry of iron into the brain is strictly controlled [27] (Fig. 1). The BBB capillary endothelium lacks fenestrations and features tight junctions, so the major way for iron transport into the cell is through transcytosis: iron ions bind with transferrin to enter the blood, and they are later released by pH changes [31]. The transferrin-iron complex binds to membrane protein transferrin receptor 1 (TFR1) on the endothelium of BBB, triggers endocytosis and forms endosomes [32]. TFR is highly expressed in the BBB. With the gradual acidification of endosomes, iron is separated from transferrin and then released into the cytosol via FPN after being reduced to Fe^2+^ by ferrireductase[28]. Transferrin is distributed into the plasma and by recirculating with TFR1, it comes back to the cytosolic membrane[29]. TFR family also plays an important role in iron metabolism: TFR1 expression is regulated at the transcription and post-transcription levels by iron regulatory proteins and iron responsive elements [30]. The same goes for the regulation of FPN expression [31]. TFR2, the homolog of TFR1, acts as an iron sensor and regulates the expression of hepcidin and thus further regulates iron metabolism [26, 32].

There are still other ways for iron transportation. Plasma non-transferrin-bound iron (NTBI) can enter the cytosol via DMT1[33], Zrt- and Irt-like protein 8 [34] or 14 [35]. However, this pathway only transports divalent metal ions, so ferrireductases, such as duodenal cytochrome b561 [36], six-transmembrane epithelial antigen of prostate [37] and prion proteins [38], are needed to reduce Fe^3+^ to Fe^2+^ in plasma. In addition, vesicles which contain transferrin or transferrin-iron complex can also be released directly on the basolateral side of the basement membrane [39]. Lactoferrin also participates in the trans-barrier transport of iron in the brain. Different from transferrin, it only releases iron in more acidic lysosomes [40-42]. Similar to the transport through BBB, iron transport across the blood-cerebrospinal fluid barrier, is another important route to enter the brain [27]. In most cases, iron is released into the brain via the FPN on the basement membrane side of the endothelium, whereas heme export can depend on the feline leukemia virus C receptor [43] and ATP binding cassette protein G2 [44].

After entering the brain, Fe^2+^ is oxidized to Fe^3+^ by ceruloplasmin on astrocyte foot process, and then Fe^3+^ rapidly binds to transferrin and is subsequently taken up by brain parenchymal cells [45-47]. Most brain parenchymal cells express TFR and they take up iron ions mainly through this pathway [48] (Fig. 1). Transferrin saturation in cerebrospinal fluid is much higher than in plasma [49, 50]. This disparity may weaken the brain’s regulation over iron when iron is overloaded and predispose brain cells to pathophysiological events such as ferroptosis. Some iron ions also bind to ferritin in the brain extracellular fluid, and then they are carried into oligodendrocytes by ferritin receptors on the membrane surface [46, 51, 52]. Other NTBI may enter brain cells (such as astrocytes) via DMT1 or trivalent cation-specific transport [53, 54]. As for the export of intracerebral iron, transferrin-bound iron can return to the systemic circulation via arachnoid granules or endothelial cells basement membrane side TFR1 [45]. However, there is a lack of evidence to confirm this process.

## 3. Mechanisms of ferroptosis during ICH

Disorders of iron metabolism, increase of lipid peroxidation and the impairment of GSH-GPX4 antioxidant systems are possible pathogenic mechanisms of ferroptosis.

### 3.1 The iron metabolism abnormalities

Iron has a strong redox activity, so changes in iron content and distribution can affect lipid peroxidation and free radical production, which can accelerate the onset of ferroptosis. The concentration of iron within a cell can be changed directly and indirectly: when hemoglobin levels in the blood change, as in the case of a brain hemorrhage, toxins of Hb are phagocytosed by microglia and nearby infiltrating macrophages and those toxins are metabolized into iron, which indirectly increases iron level [5, 55-57]; iron ions can also directly enter the cell through the forementioned pathways---transferrin receptor-mediated endocytosis and independent transport of NTBI. In the NTBI system, ferrous irons diffuse into cells by binding with the low-molecular-weight complexes, such as citrate, phosphatases, ascorbate, and peptides [58, 59]. After Fe^2+^ ions enter the cell, they are stored in the labile iron pool and can be further oxidized to Fe^3+^ by hydrogen peroxide (H_2_O_2_) through the Fenton reaction. This oxidation process generates large amounts of ROS including soluble radical, hydroxide ion and lipid alkoxy [60, 61]. If there is too much iron, microglia and macrophages may fail to completely process it and transport the excess iron out of the cell, and the surrounding neurons or astrocytes will either directly take the excess iron into the cell or indirectly through the transferrin system [45, 62]. Then iron overload leads to increased Fenton reaction and thus increased ROS production. Excessive ROS contribute to the peroxidation of adjacent lipids and attack DNA and proteins, ultimately leading to neuroinflammation [59, 63-65].

Astrocytes are key regulators of the iron metabolism in the brain, which accumulate large amounts of iron oxide nanoparticles by endocytotic mechanisms without compromising its viability [54]. It’s also interesting that hemin is toxic to cultured astrocytes while free iron is not. The reason for this discrepancy remains unknown [66, 67]. In addition, intracellular iron homeostasis and distribution are modulated by specific iron regulatory proteins [68, 69]. When iron concentrations are low, regulatory proteins bind to specific target genes, inhibit the expression of iron-binding proteins including ferritin, but increase the expression of TFRs [70]. The up-regulated TFR predisposes cells to ferroptosis [71]. Meanwhile, the activity of HO-1 is also enhanced to increase the iron content by catalyzing the breakdown of heme into Fe^2+^ [63]. The accumulation of Fe^2+^ has the pro-oxidant effect and sets the stage for ferroptosis. Together, increased iron absorption and exogenous iron supplementation, such as ferric ammonium citrate or iron chloride hexahydrate, result in iron overload [72], which can exacerbate ferroptosis. A decrease in ferritin required for iron storage may lead to free iron overload, too [73]. Xia Yi and Xiangqi Tang have recently demonstrated that ADSCs-19bM-Exos effectively alleviated the cell damage induced by hemin, improved neurologic function and inhibited ferroptosis in ICH mice [74]. Moreover, the enhancement of nuclear co-activator 4 degradation will facilitate ferritin phagocytosis and ferritin degradation, increasing free iron and ROS production [75-77].

In fact, HO-1 is both an inhibitor and promoter of ferroptosis in iron metabolism [69, 78]. On one hand, HO-1 is moderately activated when ROS are scavenged [79, 80] and it plays a cytoprotective role by degrading the free iron and biliverdin to prevent cellular senescence [81-83]; on the other hand, when HO-1 is highly activated, it plays a pro-oxidative role by accumulating more iron and producing more ROS, thus speeding up ferroptosis [63, 84]. In addition, HO-1 in the iron metabolism may influence macrophage/microglial phenotype switch and further lead to systemic inflammation development[85, 86]. Moreover, the antioxidant response is impaired as a result of the reduced NADPH levels [87]. Nrf2, an antioxidant pathway, can modulate intracellular heme synthesis and metabolism by regulating the expression of HO-1, thus it influences the heme-binding ferrous content [88-90]. It has also been proved that Nrf2 activation can upregulate the expression of genes involved in iron and ROS metabolism [91, 92].

### 3.2 Inactivation of glutathione-dependent antioxidant defenses

Redox balance is vital to maintain normal brain function. System Xc-, the cystine/glutamate antiporter, can transport an extracellular cystine molecule into the cell while transporting an intracellular glutamate molecule out of the cell [93-96]. Therefore, it plays an important role in keeping cerebral intracellular redox balance. Cystine transported into the cytosol is reduced to cysteine, which subsequently produces GSH in the presence of glutamate-cysteine ligase and GSH synthetase [97]. Glutathione peroxidase 4 (GPX4) can catalyze the reaction of GSH with lipid hydroperoxides (LOOH) to convert LOOH to non-toxic lipid alcohols, thus balancing the redox in the body [97]. Compared to the liver, the brain has a poorer endogenous antioxidant defense, which makes it more sensitive to oxidative stress and more susceptible to damage [98]. After ICH, excessive ROS produced through ferroptosis can disrupt the redox balance and cause brain damage. In the ICH rat model, the GPX4 level in the brain was reduced to the lowest level within 24 hours [99]. If GPX4 was inhibited or knocked out in advance, brain damage was more severe [99]. Meanwhile, Shaohua Wang *et al*. found that the concentration of GSH was also significantly decreased in the rat ICH model [100]. Both GSH and GPX4 are two important components of the biological antioxidant system, and each of their reduction may promote ferroptosis. This hypothesis has been verified outside the brain. Yun Sun *et al.* used buthionine sulphoximine to hinder the de novo synthesis of GSH and they observed ferroptosis [101]. Other GSH synthesis blockers, acetaminophen and sorafenib loaded manganese-silica nanodrugs, have the same effect of inducing ferroptosis [102, 103]. Xinbing Sui *et al.* used RAS-selective lethal 3 to suppress GPX4, and this suppression of GPX4 promoted ferroptosis[104]. Jose Pedro Friedmann Angeli *et al*. compared the renal functions in mice before and after the knockdown of GPX4 and genetically confirmed the important role of GPX4 in ferroptosis [105]. Dixon *et al*. found that Erastin could directly inhibit System Xc- and they proposed the concept of ferroptosis [72]. Sulfasalazine, sorafenib and extracellular excess of Glu also inhibit System Xc- and promote ferroptosis production [106-110]. In addition, radiotherapy and immunotherapy targeting System Xc- have emerged as novel therapies for ferroptosis [111, 112]. Xueting Lang *et al.* reported that radiotherapy and immunotherapy synergistically inhibited SLC7A11, the light chain of System Xc- [111]. Weimin Wang *et al.* also reported that CD8+ T cells could downregulate the expression of System Xc- heavy chain SLC3A2 [112], thereby promoting ferroptosis. After erastin inhibits System Xc-, intracellular cysteine-raw material for GSH synthesis---is further reduced [113], followed by GPX4 dysfunction which breaks the balance between intracellular oxidation and anti-oxidant system and triggers ferroptosis [113-115]. Erastin also blocks the mitochondrial voltage-dependent anion channel, which impairs mitochondrial function and produces excessive ROS [116, 117]. Minghui Gao *et al.* showed that mitochondria are essential for ferroptosis induced by cysteine deficiency, but have little effect in ferroptosis induced by GPX4 inhibition [118]. Therefore, whether the mitochondrial pathway can initiate ferroptosis or not remains controversial and needs further investigation.

### 3.3 Iron-dependent lipid peroxidation: the essence of ferroptosis

Lipid peroxidation is a process in which oxidants, such as free radicals, attack lipids containing carbon-carbon double bonds, particularly polyunsaturated fatty acids (PUFA) [119], through enzymatic or non-enzymatic pathways [120]. Due to the active metabolism in brain and the high content of PUFAs in membrane phospholipids [121], neurons are easy to be oxidized, which sets the stage for lipid peroxidation under abnormal conditions. After ICH, the ruptured erythrocytes release hemoglobin/heme/iron, and excess free iron (Fe^2+^) reacts with H_2_O_2_ and generates a range of ROS, including soluble hydroxyl groups and lipid alkoxyl radicals, through this non-enzymatic Fenton reaction [119]. When the body's antioxidant system fails to remove overloaded ROS, excessive ROS will attack brain biofilm phospholipids and trigger lipid peroxidation. Then, the peroxidation of PUFAs generates various toxic oxidation products, including LOOH, malondialdehyde (MDA) and 4-hydroxynonanal (4-HNE). It has been proved that lipid peroxidation could reduce membrane stability and fluidity, alter the biological activities of membrane-associated proteins, and induce infiltration of many substances that affect cell activity and survival [122]. ROS can also attack DNA and proteins, oxidize bases, break single-strand, and inhibit critical DNA repair pathways. MDA, a toxic product of lipid peroxidation, is one of the most commonly used and the most reliable markers for clinical detection of oxidative stress and ferroptosis [123]. Its excessive accumulation is associated with many diseases, including cancer and central nervous system diseases such as Alzheimer's disease [124]. Another toxic product of lipid peroxidation, 4-HNE, is a signal molecule. It regulates several stress-sensitive transcription factors, such as Nrf2, affects cell proliferation and/or differentiation, and participates in several cell death pathways including autophagy, necrosis and apoptosis, so as to jointly affect disease development [119].

## 4. Treatments of ICH through ferroptosis pathways

Ferroptosis in ICH is closely related to iron abnormality, GSH-dependent antioxidant defenses and lipid peroxidation, and these pathological events can activate inflammatory response and cause neuronal lesions. Thus, treatments targeting these ferroptosis-related changes provide novel ideas for inhibiting ferroptosis, preventing neuroinflammatory injury, and restoring neural function (Table 1).

**Table 1:** Treatments of ICH through ferroptosis pathways.

Drugs	Year	Object	Mechanisms	Impact/Effect
**(-)-Epicatechin [129]**	2014	Male and female ICH mice.	Diminishing heme oxygenase-1 induction and brain iron deposition via an Nrf2-independent pathway.	Reducing lesion volume, improving neurological deficits and rescuing neuronal degeneration after cerebral hemorrhage.
**Dopamine [132]**	2016	Erastin-induced cell death in cancer and non-cancer cell lines.	Partly suppressing erastin-induced GPX4 protein degradation.	Playing a newly discovered role in the inhibition of erastin-induced ferroptosis in both cancer and non-cancer cells.
**VK-28 [10]**	2017	An ex vivo OHSC model and an in vivo collagenase-induced ICH mice model.	Polarizing microglia/macrophages toward an M2-like phenotype, attenuating ROS production and iron deposition after ICH.	Decreasing cell death and microglial activation around hematoma and improving neurologic function.
**Edaravone [140]**	2018	FeCl_3_ injection rats ICH model.	Inhibiting oxidative stress and lipid peroxidation, activating Nrf2/HO-1 signal pathway.	Reducing brain edema and ventricular dilatation and improving the ability of learning and memory after ICH.
**N-acetylcysteine [201]**	2018	Hemorrhagic stroke mice model.	Targeting toxic lipids derived from 5- LOXs and increasing glutathione levels.	Reducing neuronal death after injury and promoting functional recovery.
**Minocycline [125]**	2019	Aged female ICH rats.	Chelating iron in cortical neuron, reducing ICH-induced iron deposition, and downregulating iron handling protein.	Relieving brain swelling, neuroinflammation, neuronal loss, delayed brain atrophy and neurological deficits.
**Selenium [202]**	2019	Collagenase-induced ICH mouse model.	Augmenting GPX4 and other genes in the transcriptional program via coordinated activation of TFAP2c and Sp1.	Protecting neurons and improving behavior.
**Glutathione [131]**	2020	Autologous blood injection induced ICH mice model.	Up-regulating protein expression of complex I.	Suppressing the aggravation of neurological deficits, attenuating injury of brain edema and disruption of blood-brain barrier.
**Fer-1 [143]**	2021	Post-subarachnoid hemorrhage rat model.	Scavenging of alkoxyl radicals produced by ferrous iron from lipid hydroperoxides.	Reducing blood-brain barrier damage, brain edema, behavioral defect and neuronal damage.
**PIH [11]**	2021	ICH mouse model.	Reducing ROS production, iron accumulation, and lipid peroxidation around the hematoma peripheral tissue.	Protecting mice against hemorrhage stroke, mitigating inflammation and ferroptosis.

Abbreviation: Fer-1, Ferrostatin-1; GPX4, glutathione peroxidase 4; ICH, intracerebral hemorrhage; LOX, lipoxygenase; OHSC, Organotypic hippocampal slice culture; PIH, Pyridoxal isonicotinoyl hydrazine; ROS, reactive oxygen species.

### 4.1 Treatments targeting iron abnormality

ICH triggers intracellular iron overload, which in turn leads to the onset of ferroptosis. Thus, it is especially important to reduce the iron level right after ICH. The efficacy of iron chelators, such as deferoxamine, minocycline and VK-28, in the treatment of cerebral hemorrhage has been demonstrated in preclinical studies [10, 125, 126]. These iron chelators reduce the accumulation of iron around the hematoma. An increasing number of new nanomaterials have emerged to improve ferroptosis. For example, DEF-HCC-PEG can alleviate the toxicity of excess hemoglobin and iron [83]; The porous selenium@SiO2 nanocomposite can exert neuroprotective effects by restraining oxidative stress, and it serves as a potential method for clinical treatment of oxidative stress-related brain injuries beyond ICH [127]. Besides iron chelators and nanomaterials, various other kinds of drugs have been recently studied to alleviate injuries after ICH: baicalin, studied in ICH model mice, was reported to inhibit ferroptosis[128]; PIH, a lipophilic iron-chelating agent, reduces iron accumulation, ROS production and lipid peroxidation around the hematoma peripheral tissue [11]; (-) Epicatechin, a brain-permeable flavanol, reduces lesion volume and improves neurological deficits by depositing brain iron, activating Nrf2 signaling pathway and inducing HO-1 [129].

### 4.2 Treatments targeting GSH-dependent antioxidant defenses

N-acetylcysteine (NAC), a prodrug of cysteine, acts on System Xc-, promotes the accumulation of cysteine in neuronal cells, increases the amount of the antioxidant GSH and thus resists ferroptosis [130]. When NAC is combined with Prostaglandin E2, its therapeutic effect remains unchanged despite a decrease in its dose [130]. Direct administration of GSH serves as a potential treatment for ICH: Xiaojun Diao *et al.* found that GSH treatment in ICH mice model attenuated neurological damage [131]. Enhancement of GPX4 level and activity also provides a treatment for ICH: Zhuwei Zhang *et al*. found that overexpression of GPX4 effectively increased the level of GPX4 and significantly alleviated neuronal dysfunction after ICH [99]; Ding Wang *et al*. found that dopamine partly suppressed erastin-induced GPX4 protein degradation, which in turn enhanced GPX4 stability [132]. The transcription factor Nrf2 can upregulate both GSH and GPX4 levels, effectively preventing ferroptosis [133, 134]. In addition, selenium is an essential micronutrient for GPX4 synthesis by driving GPX4 transcriptional expression [135]. Ishraq Alim *et al*. found that intracerebroventricular or systemic administration of selenium could activate adaptive transcriptional responses to ferroptosis, thereby improving functional recovery after stroke [136]. Moreover, the downregulation of ATF4 activation can counteract glutamate analog-induced ferroptosis, which in turn alleviates the brain damage after ICH [134, 137]. To conclude, therapies targeting GSH-dependent antioxidant defenses, including enhancing the transport activity of Xc-, increasing the amount of GSH, and promoting both level and activity of GPX4, open new windows for ICH treatment.

### 4.3 Treatments targeting lipid peroxidation

ICH-induced ROS can trigger ferroptosis via lipid peroxidation and further lead to secondary brain injury. Thus, the inhibition of lipid peroxidation to prevent ferroptosis may provide a potential treatment for ICH. Since lipid peroxidation depends on enzymatic or non-enzymatic pathways, treatment strategies can be categorized into two kinds: lipid autooxidation inhibitors (such as Radical-Trapping Antioxidants, RTAs) and lipoxygenase (LOX) inhibitors [138]. Some rat experiments proved that oxygen-free radical scavenger Edaravone can inhibit lipid peroxidation by increasing antioxidant superoxide dismutase and decreasing lipid peroxidation product MDA. Thus, Edaravone can protect ependymal cilia and neurons from oxidative stress, reduce brain edema and ventricular dilatation in the intraventricular hemorrhage model or FeCl_3_ injection model, and improve the ability of learning and memory after ICH [139, 140]. In addition, many studies have shown that ferrostatin-1 (Fer-1), an outstanding RTA in phospholipid bilayers [141], can also prevent lipid peroxidation by scavenging the initiating alkoxyl radicals generated by Fe^2+^ [142]. The treatment of Fer-1 in rats with subarachnoid hemorrhage could increase the expression of SLC7A11 and GPX4 and further alleviate the damage of BBB, brain edema, behavioral defect and neuron injury [143]. As an important part of lipid peroxidation, oxidation of PUFA by LOXs promotes LOOH production [144]. Yu Liu *et al.* reported that inhibition of 12/15-LOX reduced hemorrhagic transformation in experimental stroke mice [145]. NAC, the aforementioned participant in GSH-dependent antioxidant defenses, also targets toxic lipids derived from 5-LOX and increases GSH levels to prevent ferroptosis and improve prognosis in hemorrhagic stroke mice[130]. In addition, vitamin E hydroquinone, an endogenous modulator of ferroptosis, plays an antioxidant role by regulating 15-LOX [146]. Therefore, lipid autooxidation inhibitors and LOX inhibitors may become potential strategies for ICH treatment in the future.

## 5. Other ferroptosis related pathways in ICH pathology

Ferroptosis works in concert with other mechanisms including immune regulation, oxidative stress, and microRNA intervention in the pathogenesis of cerebral hemorrhage. To further explore their interactions, we are going to elucidate the role of ferroptosis in different brain cells and different organelles after ICH.

### 5.1 Different organelles in the ferroptosis after ICH

Ferroptosis integrates multiple pro-survival or pro-death signals from subcellular organelles. When ferroptosis occurs, the cell keeps normal membranes and nuclei, but its mitochondria undergo some ultrastructural changes, such as crinkled morphology [72, 147, 148]. Mitochondria are the power plants in a cell and their damages can affect the normal physiological activities of the body. For example, neurons are highly sensitive to mitochondrial lesions because they are particularly dependent on mitochondrial ATP production and calcium buffer[149]. Qian Li *et al.* found that both collagenase-induced acute and subacute models of ICH in mice displayed crinkled mitochondria [150]. However, in the chronic phase model, no consistent increase in the number of crinkled mitochondria was observed. Instead, the number of swollen mitochondria increased [150], suggesting that there are other types of cell death beyond ferroptosis after ICH. Mitochondrial damage in ferroptosis has also been related to neurodegenerative diseases, such as Alzheimer's Disease, Parkinson's disease, and amyotrophic lateral sclerosis [151-154].

Mitochondria are also the major source of endogenous ROS. Excessive iron accumulation after ICH may increase intracellular ROS production [8, 155]. Excess ROS attack sensitive fatty acids and facilitate lipid peroxidation, which impairs lipid membrane integrity and mitochondrial function, predisposing the cell to ferroptosis [148]. Huang *et al.* found that continuous iron exposure in human dopaminergic neuroblastoma SH-SY5Y cells elevated mitochondrial ROS levels [156]. Ilaria Pelizzoni reported that clearance of mitochondrial ROS prevented injury to the hippocampus from iron overexposure and maintained the integrity of mitochondrial morphology and membrane potential [157]. In addition to ferroptosis, activation of mitochondrial permeability transition pores (mPTP) may also underlie the occurrence of neural injury after ICH [158]. ROS derived from the ferroptosis and other death pathways after ICH, can attack the mPTP [158, 159]. Once the mPTP is activated, water, macromolecules and iron ions will flood into the mitochondrial matrix, which destroys the mitochondrial respiratory chain and consequently triggers cell death [155, 160, 161]. However, mROS is not a necessity for ferroptosis and it remains controversial whether the mitochondrial pathway is an initiating factor for ferroptosis or not. Within minutes of arterial occlusion in ischemic stroke, mitochondrial membrane will depolarizate and ATP production collapse within the neuronal cell, triggering ischemic cascades such as membrane ion pump failure and plasma membrane depolarization [161, 162]. Furthermore, Isaac García-Yébenes *et al.* found that in animals with cerebral ischemia, iron overload facilitates the conversion from transient ischemic strokes to hemorrhagic strokes in animal models [163].

The endoplasmic reticulum (ER) is also related to ferroptosis after ICH. Some studies have suggested an association between ER stress and ferroptosis. ER stress is mainly caused by unfolded protein response (UPR)-the accumulation of misfolded proteins in the ER which disrupts ER homeostasis [164]. The ER stress signal is initiated by three effector proteins: ATF6, the protein kinase RNA-like endoplasmic reticulum kinase (PERK), and inositol-requiring protein-1 [165]. ICH increases free hemoglobin in brain tissue, and they are further degraded into heme. Interestingly, Tamás Gáll *et al.* showed that those hemes can increase ER stress markers in human aortic plain muscle cells [166]. ER stress also increases the expression and activity of GPX4, a molecule in GSH-dependent antioxidant defenses, through the activation of PERK/ATF4 axis [167]. Moreover, RNA sequencing showed the System Xc- inhibition, followed by up-regulation of cation transport regulator homolog 1 and concomitant ER stress initiation [168]. Redox imbalance and lipid peroxidation in ferroptosis may also trigger ER stress [169]. However, studies on the relationship between ER stress and ferroptosis in ICH are still scant. Nowadays, most of the studies on ferroptosis are focused on its role in cancer. Given the similarities of the ferroptosis mechanism between neurons and cancer cells, we can draw lessons from cancer cell studies to better study ferroptosis in neurons after ICH [137].

Lysosome, a member of the intracellular membrane system, has also been demonstrated in recent studies to participate in ferroptosis. Lin Li *et al.* have shown that carboxyl-modified polystyrene nanoparticles could induce nuclear translocation of transcription factor EB through lysosomal stress, which decreases cellular ROS and inhibits ferroptosis [170]. MDA, the final product of lipid peroxidation, was detected in the activation of lysosome-dependent cell death and it promoted erastin-induced ferroptosis in human pancreatic ductal adenocarcinoma cell lines, which indicates the important role of lysosomal lipid peroxidation in ferroptosis [171].

### 5.2 Neuroglial cells and neurons in the ferroptosis after ICH

ICH occurs when blood vessels rupture and then the broken erythrocytes release numerous hemoglobin/heme [172, 173]. Typically, these toxins are phagocytosed by microglia and nearby infiltrating macrophages, and they are metabolized into iron by these cells, which produces more ROS and triggers lipid peroxidation [5, 55-57]. In this conversion from heme to iron, HO plays a crucial role. Two active isomers of HO, HO-1 and HO-2 are found in the brain. HO-1, also known as heat shock protein 32, is poorly expressed by neurons but highly expressed by microglia and astrocytes; while HO-2 is highly expressed by neurons [69, 174]. A study has implied that ICH could induce more HO-1 production which leads to iron overload [175]. As a downstream reaction to iron overload, a large number of ROS can be formed through the Fenton reaction with the presence of H_2_O_2_, and these radicals attack the cell membrane, DNA, and proteins, triggering secondary damage to the cell, especially neuroinflammation [64, 65]. Some studies have shown that there is an increase of DMT1 and FPN in ICH rats, negatively correlating with the amount of Fe^2+^ in cells [69, 176]. Fpn1 overexpression increases iron efflux and acts as a cytoprotective agent while DMT1 plays the opposite role---it enhances the influx of iron into the cell [177]. Moreover, the contents of ferritin, transferrin, TFR, and HO-1 in the brain increase significantly after ICH [125, 178].

GPX4, mainly expressed in oligodendrocytes and neurons, plays an indispensable role in neuronal ferroptosis. It is mainly located in the cytoplasm of neurons and the nucleus of oligodendrocytes [179]. GPX4 prevents lipid peroxidation by converting reduced GSH to GSH disulfide and lipid hydroperoxides or H_2_O_2_ to the corresponding alcohols and/or water [105, 180]. In fact, the level of GSH in astrocytes is higher than that in oligodendrocytes, so the GSH/GPX4 system may fail to completely inhibit lipid peroxidation [79, 181]. Furthermore, the total amount of iron in oligodendrocyte precursor cells is much higher than that in astrocytes. Together these features make oligodendrocytes more susceptible to ferroptosis, and even may aggravate demyelination [182]. Microglia are the main phagocytes in the brain, and they play a crucial role in ferroptosis. When the brain is under injuries such as ischemic stroke or ICH, microglia cells will undergo a specific phenotype change [183, 184]. However, despite many relevant studies, the question that whether microglia undergo M1 or M2 polarization still has no answer yet [185]. M1 and M2 cells have different resistance to ferroptosis: The depletion or inactivation of the inducible nitric oxide synthase in the activated M1 macrophages/microglia predisposes M1 cells to ferroptosis, while NO-donors strengthens the resistance of M2 cells to ferroptosis [186]. Microglia also serves as a bridge linking neuroinflammation and ferroptosis. Neuroinflammation decreases ferritin and increases DMT1 in the hippocampus, promoting iron uptake and output [187, 188]. And this inflammatory response mainly takes place in microglia because the iron accumulation induced by inflammatory stimuli and hepcidin only takes place in neurons and microglia [189, 190]. To conclude, different brain cells participate in ferroptosis through different mechanisms.

### 5.3 Connections between ferroptosis and other cell death pathways after ICH

ICH is a complicated brain disease regulated by many cell death pathways, including ferroptosis, apoptosis, autophagy, necroptosis, eryptosis, oxytosis and so on. A growing number of investigations have shown that ferroptosis features unique ultrastructural characteristics, molecule mechanism and outcomes compared with other types of programmed cell death [191]. However, the underlying connections between ferroptosis and other types of cell death after ICH have not been thoroughly explored.

The brain injuries after ICH include:1)the space occupation and mechanical deformation of brain tissue caused by hematoma [192]; 2)the secondary injury or death of nerve cells caused by inflammatory factors activation, hemoglobin and free iron accumulation. As we mentioned above, excessive ROS produced by iron overload can attack the cell membrane and organelle membrane of nerve cells around hematoma and trigger the process of iron-dependent lipid peroxidation. At the same time, lipid peroxidation products can induce apoptosis and autophagy in different mechanisms [193]. This shows that lipid peroxidation bridges ferroptosis with apoptosis and autophagy. What’s more, ferroptosis accelerates inflammation through immunogenically releasing damage-associated molecules and alarmins [194]. And then, those inflammatory factors, as activators, can initiate other cell death pathways such as necroptosis, apoptosis, pyroptosis through different signal pathways [193]. Therefore, inflammation may be another bridge between ferroptosis and other cell death pathways. A more specific example is mTOR, a key molecule in cellular autophagy after ICH. Upregulation of mTOR attenuates autophagy in microglia, thereby reducing secondary inflammatory damage [195, 196]. There is also a close connection between ferroptosis and necroptosis. By using HO-1 as a marker to track hemin and using immunofluorescence techniques to label necrosomes, it was observed that hemin may directly induce neuronal injury through RIPK1 and RIPK3 signal pathways of necroptosis [197]. Moreover, the recently defined eryptosis is a novel pathway for the programmed suicide death of erythrocytes, and eryptosis is also important in ICH as the broken erythrocytes will release iron and hemin [7, 198, 199]. Oxytosis and ferroptosis also share lots of similarities and oxytosis is even considered an integral part of ferroptosis [73, 200]. In summary, more and more studies have shown that ferroptosis is often concomitant with other types of cell death. Ferroptosis, together with other cell death pathways, may fully elucidate the pathological changes in cells after ICH and a combined therapy may become an effective and novel treatment for ICH.

## 6. Conclusions and prospects

Ferroptosis is a newly discovered form of programmed cell death in recent years. Ferroptosis involves many pathways: homeostasis disruption of iron and iron regulatory proteins; glutathione-dependent antioxidant defense system dysregulation; iron-dependent lipid peroxidation. However, it is important to note that these three pathways are not completely independent of each other, but are intricately orchestrated to mediate ferroptosis. Different organelles, such as mitochondria, ER and lysosome, also participate in ferroptosis. Scientists have observed mitochondrial contraction and mitochondrial outer membrane rupture under the electron microscope, and they consider this phenomenon as one of the most likely causes of ferroptosis. Ferroptosis is also linked to other forms of programmed cell death, including apoptosis, autophagy, necroptosis, eryptosis and oxytosis. Together they mediate subsequent tissue damage and many pathological processes.

Recently, the mechanisms of ferroptosis in cancer are under intense research. As there are close correlations between cancer and ICH, we can draw some lessons from research in cancer and apply them to ICH research. The occupying effect of the hematoma after ICH can cause primary damage to the brain, followed by lysis of red blood cells and accumulation of heme and iron induced ferroptosis. Heme and iron can induce large amounts of hydroxyl radicals through lipid peroxidation or Fenton reaction. Those radicals attack the cell membrane of peripheral nerve cells and organelles membrane of mitochondria and endoplasmic reticulum, and they further disrupt local brain metabolism and cause severe secondary damage to the brain. Current treatments of ICH mainly target symptomatic therapies such as hemostasis, antihypertension, dehydration to lower cranial pressure, and surgical treatment, while there are few treatments targeting the pathological mechanisms of ICH because of the lack of relevant research and unsolved challenges. There are some studies on ICH treatments targeted at ferroptosis. They reported certain drugs that can alleviate lipid peroxidation (e.g., Fer-1), that can rescue iron metabolism disruption (e.g., DEF) and that can strengthen GSH-dependent antioxidant defenses (e.g., NAC) after ICH. However, the effectiveness of these treatments has not been confirmed. Future research will continue to explore the mechanisms of ferroptosis and figure out its interaction with other pathological processes. Through the in-depth exploration into ferroptosis, researchers can develop novel and effective treatments for ICH and many other intracerebral diseases.
